# Supplementary material for: Comparison of Clinical Outcomes, Risks, and Costs for 20,910 Donor In Vitro Fertilization and 16,850 Donor Artificial Insemination Treatment Cycles: A Retrospective Analysis in China
Source: J Clin Med. 2023 Jan 26;12(3):954. doi: 10.3390/jcm12030954 (PMC9917547; doi:10.3390/jcm12030954)

Figure legends

Figure S1 Cycles included in the study.

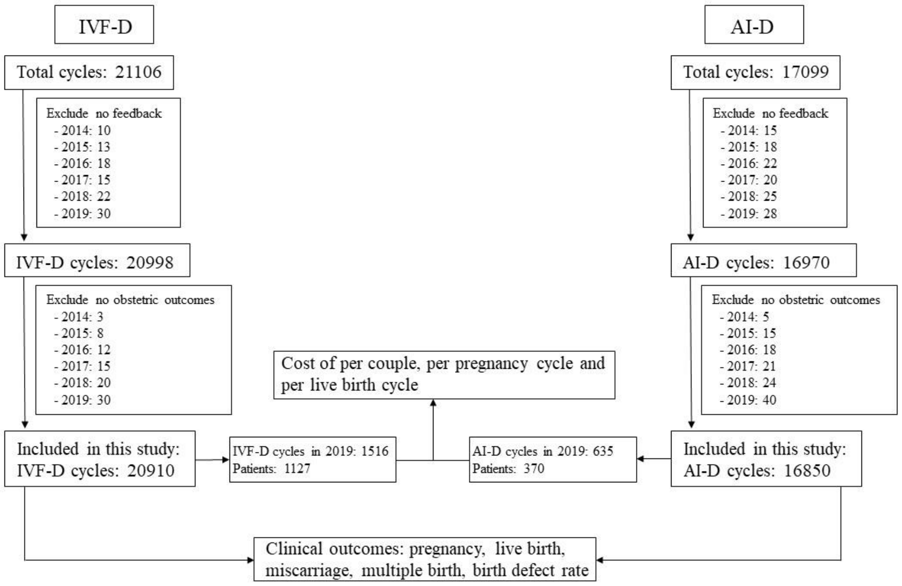

Figure S2 Cycle changes of IVF-D and AI-D treatments.

(A) Cycle changes of IVF-D and AI-D treatments in CITIC-Xiangya. (B) Cycle changes of ART treatment in CITIC-Xiangya. (C) Cycle changes of IVF-D and AI-D treatments in other reproductive centers.

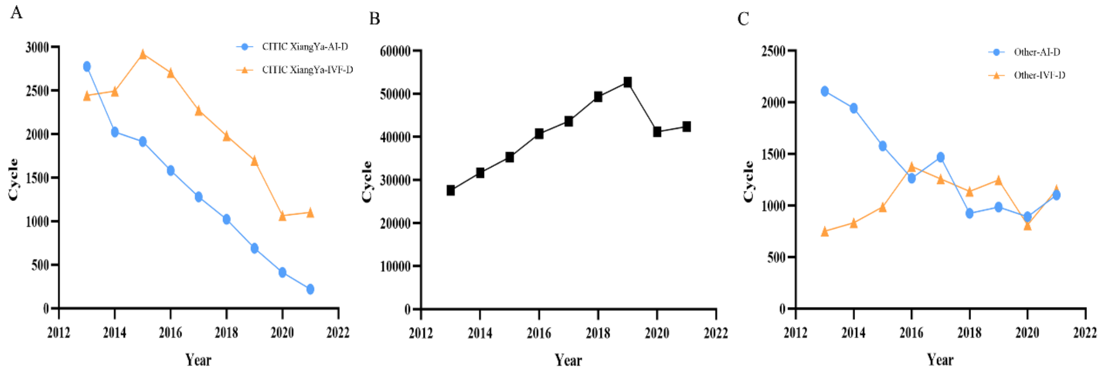

Figure S3 Distribution of sperm donation services of Reproductive and Genetic Hospital of CITIC-Xiangya(Hunan Province Human Sperm Bank).

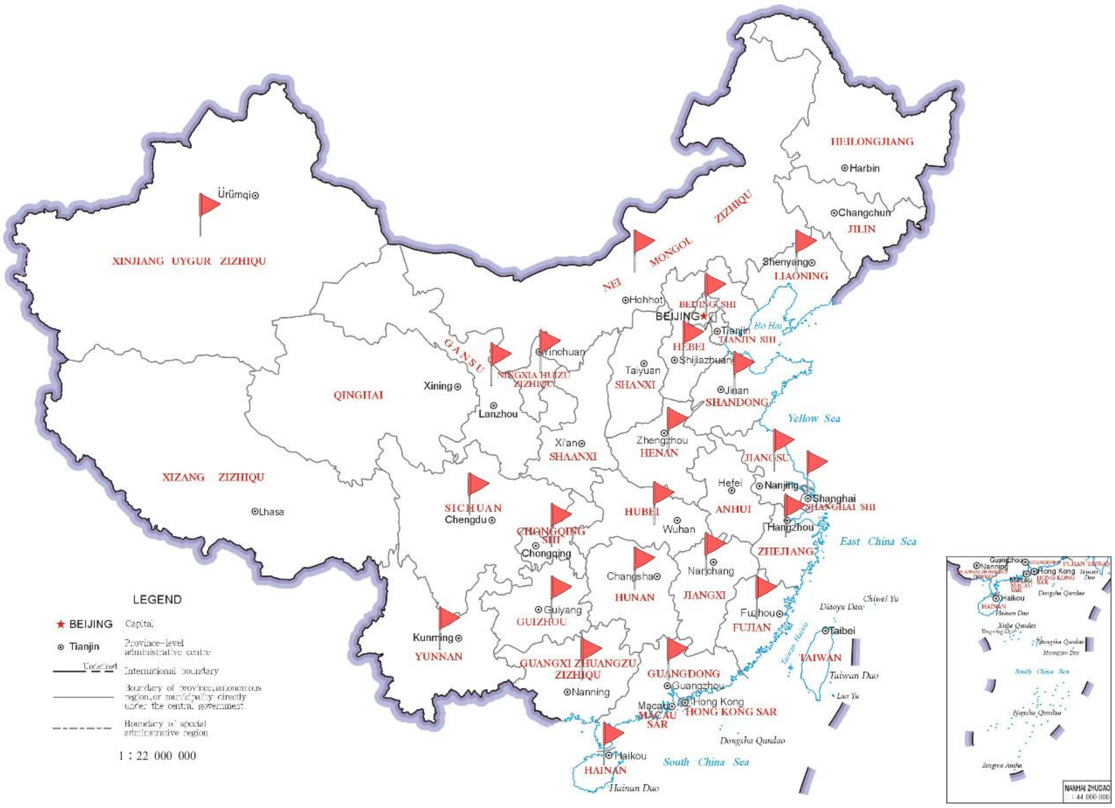

Supplement: Supplementary file 1 [file jcm-12-00954-s001.zip › jcm-2136009-supplementary.pdf]
